# Transcriptional Regulation of the Human 5-HT1A Receptor Gene by Lithium: Role of Deaf1 and GSK3β

**DOI:** 10.3390/ijms242115620

**Published:** 2023-10-26

**Authors:** Emerson F. Harkin, Georges Nasrallah, Brice Le François, Paul R. Albert

**Affiliations:** Ottawa Hospital Research Institute (Neuroscience), University of Ottawa, 451 Smyth Road, Ottawa, ON K1H-8M5, Canadabrice.lefrancois@dnagenotek.com (B.L.F.)

**Keywords:** lithium, serotonin, receptor, GSK3, repressor, phosphorylation

## Abstract

Serotonin 1A (5-HT1A) autoreceptors located on serotonin neurons inhibit their activity, and their upregulation has been implicated in depression, suicide and resistance to antidepressant treatment. Conversely, post-synaptic 5-HT1A heteroreceptors are important for antidepressant response. The transcription factor deformed epidermal autoregulatory factor 1 (Deaf1) acts as a presynaptic repressor and postsynaptic enhancer of 5-HT1A transcription, but the mechanism is unclear. Because Deaf1 interacts with and is phosphorylated by glycogen synthase kinase 3β (GSK3β)—a constitutively active protein kinase that is inhibited by the mood stabilizer lithium at therapeutic concentrations—we investigated the role of GSK3β in Deaf1 regulation of human 5-HT1A transcription. In 5-HT1A promoter-reporter assays, human HEK293 kidney and 5-HT1A-expressing SKN-SH neuroblastoma cells, transfection of Deaf1 reduced 5-HT1A promoter activity by ~45%. To identify potential GSK3β site(s) on Deaf1, point mutations of known and predicted phosphorylation sites on Deaf1 were tested. Deaf1 repressor function was not affected by any of the mutants tested except the Y300F mutant, which augmented Deaf1 repression. Both lithium and the selective GSK3 inhibitors CHIR-99021 and AR-014418 attenuated and reversed Deaf1 repression compared to vector. This inhibition was at concentrations that maximally inhibit GSK3β activity as detected by the GSK3β-sensitive TCF/LEF reporter construct. Our results support the hypothesis that GSK3β regulates the activity of Deaf1 to repress 5-HT1A transcription and provide a potential mechanism for actions of GSK3 inhibitors on behavior.

## 1. Introduction

Major depressive disorder (MDD) is a debilitating psychiatric disorder characterized by persistent low mood and loss of pleasure [1]. MDD has a lifetime prevalence of 16% [2] and is a leading contributor to the global burden of disease [3]. Both basic research and clinical strategies have increasingly emphasized the role of serotonin in depression [4,5,6], despite inconsistencies in clinical studies. This is partly because of the abundance of evidence connecting abnormalities in the serotonin system to depression in humans [7,8,9,10,11] and because of the overall efficacy of selective serotonin reuptake inhibitors (SSRIs) in the treatment of depression [12,13]. However, most patients must try multiple treatments to relieve their depressive symptoms [12]. A better understanding of the regulation of the serotonin system may help identify responsive patients and new treatment options.

Over the past decade, animal studies have increasingly implicated the serotonin system in processing emotionally salient stimuli [14,15,16], further supporting the involvement of serotonin in the pathogenesis and treatment of depression; recent work is beginning to shed light on how this processing occurs [17]. A key regulator of the activity of the serotonin system is the 5-HT1A receptor. Presynaptic 5-HT1A autoreceptors are expressed on 5-HT neurons and powerfully inhibit their firing activity through negative feedback following 5-HT release [18]. Elevated expression of 5-HT_1A_ autoreceptors is associated with an increased risk of depression [9,19,20], suicide attempt [21], and reduced response to SSRIs [22]. On the other hand, 5-HT1A heteroreceptors located on non-5-HT neurons are particularly enriched in the lateral septum, hippocampus and prefrontal cortex (PFC) and are implicated in mediating the antidepressant actions of SSRIs [23,24].

Factors that differentially regulate pre- and post-synaptic 5-HT1A receptor expression are attractive drug targets to enhance antidepressant efficacy. The transcription factor deformed epidermal autoregulatory factor 1 (Deaf1, also known as NUDR) is one such protein. Deaf1 regulates 5-HT1A expression by binding to a recognition site located in the 5-HT1A promoter region [25] via its SAND (Sp100, AIRE-1, NucP41/75, DEAF1; aa 188–254) domain [26]. This site contains a functional single nucleotide polymorphism known as C(-1019)G; when the G-allele is present, Deaf1 is unable to regulate 5-HT1A promoter activity [25]. Interestingly, the 5-HT1A G-allele has been associated with depression and suicide in humans, as well as with resistance to antidepressant response [27,28,29]. Deaf1 acts as a pre-synaptic repressor and post-synaptic enhancer of 5-HT1A expression [30,31]; thus, a drug that activates Deaf1 would reduce 5-HT1A autoreceptors and enhance 5-HT1A heteroreceptors to promote the antidepressant response.

To identify regulators of Deaf1 activity, interacting proteins provide a useful lead. Interestingly, a yeast two-hybrid screen of proteins in the mTOR pathway implicated in antidepressant actions [32,33] identified Deaf1 as a glycogen synthase kinase 3β (GSK3β) interacting protein [34]. GSK3β is a constitutively active serine/threonine kinase that phosphorylates Deaf1 in vitro [34]. It is also an established target of lithium, the gold-standard for the treatment of both manic and depressive symptoms of bipolar disorder [35]. Lithium is also known to augment the antidepressant actions of ketamine [36,37]. Although acute effects of any drug are unlikely to result from changes in gene transcription, it is worth noting that another specific inhibitor of GSK3, AR-014418, has shown antidepressant-like effects in the forced-swim test [38]. Together, these observations point towards GSK3β as a possible target for the treatment of mood disorders. Thus, we addressed whether GSK3β is implicated in the transcriptional regulation of the 5-HT1A receptor via Deaf1. We find that pharmacological inhibition of GSK3 attenuates Deaf1 mediated repression with the emergence of Deaf1 enhancer activity, providing a novel potential mechanism for GSK3β actions on behavior.

## 2. Results

### 2.1. Deaf1 Phosphorylation Sites

**Figure 1:**
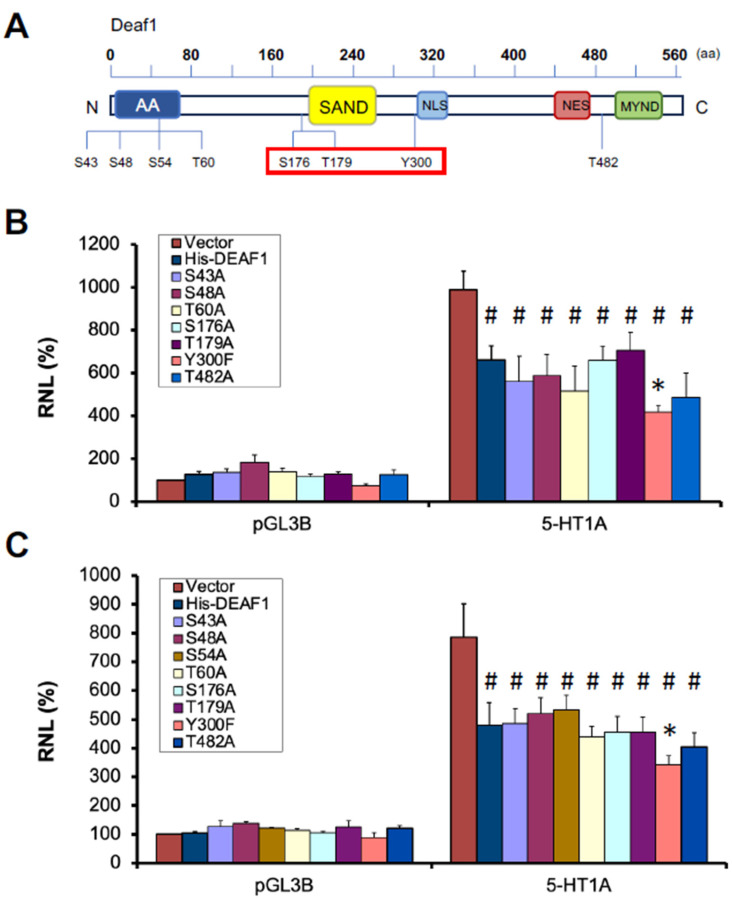
Activity of point mutants of reported and predicted Deaf1 phosphorylation sites. (**A**) Schematic diagram of the structural domains characterized in Deaf1 and positions of reported and predicted phosphorylation sites. Domains are the Alanine-Acidic domain (AA), the SAND domain (SAND), the nuclear localization signal (NLS), the nuclear export signal (NES) and the MYND domain (MYND). Previously published phosphorylated sites are framed in red. (**B**,**C**) Deaf1 and mutant activity at the 5-HT1A promoter in HEK-293 (**B**) or SKN-SH (**C**) cells. Cells were transfected with the pGL3B reporter construct (with or without the 5-HT1A promoter), the pCDNA3 construct (with or without HisDeaf1 or mutants), and pCMV-β-gal. Results for reported sites and predicted sites are both shown. Data are shown as mean ± SEM of relative normalized luminescence (RLN), i.e., the relative luciferase activity was normalized to pGL3B-Vector. Values of all samples were compared by two-way ANOVA followed by Bonferroni-adjusted pairwise comparisons to find *p* values. * *p* < 0.05 compared to HisDEAF1; ^#^ *p* < 0.01 compared to pCDNA3.

Previous work has shown that Deaf1 interacts with and is phosphorylated by GSK3β [34]. Putative phosphorylation sites on Deaf1 were screened by database screen, ranked and used to generate Deaf1 point mutants (Section 4). Deaf1 sites with demonstrated phosphorylation were identified using the Human Protein Reference Database, 2009 update [39]. Deaf1 has three major published identified phosphorylated sites that include S176 [40,41], T179 [40] and Y300 [42] (https://www.phosphosite.org/ accessed on 12 July 2023). S176 and T179 phosphorylation was identified in large-scale cation exchange chromatography experiments [40,41], while Y300 phosphorylation was found in large-scale electron transfer dissociation experiments [42]. We also identified several predicted phosphorylation sites. Interestingly, T171, S176 and T179 lie in a proline-rich sequence **T**PGPQ**S**PP**T**P similar to the GSK3 phosphorylation consensus motif (www.phosphonetworks.org accessed on 12 July 2023) **S**PPP**S/T**PPX**S**/**T**P and is located immediately adjacent to the DNA-binding SAND domain of Deaf1 (Figure 1A). To address the functional consequences of the phosphorylation of these residues, mutations were introduced in a HisDeaf1 construct. To abolish potential phosphorylation, serine and threonine residues were mutated to alanine, while tyrosine was mutated to phenylalanine. The repressor activity of the mutants was examined in HEK293 cells co-transfected with 5-HT1A-luciferase reporter construct (Figure 1B). Of the eight mutants generated, only the Y300F mutant showed altered repressor activity at the 5-HT1A promoter when compared to wild-type HisDeaf1, with increased activity. We also examined several other mutants predicted from phosphosite algorithms (Section 4) and found that none affected His-Deaf1 activity. All mutants were also tested in the SKN-SH neuroblastoma cell line and again only the Y300F mutant showed increased repressor activity at the 5-HT1A promoter (Figure 1C). Thus, none of the serine/threonine mutants altered Deaf1 repressor activity.

**Figure 2:**
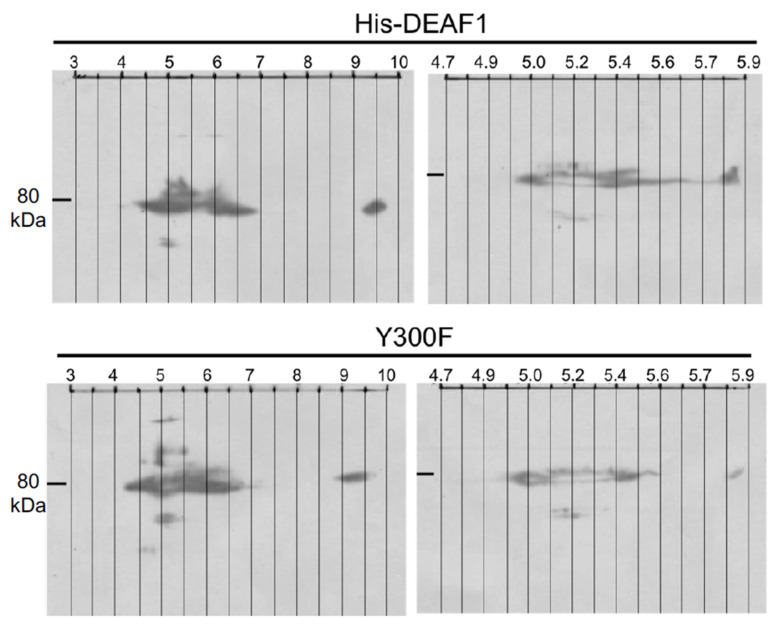
Two-dimensional-gel analysis of Deaf1 and Deaf1 Y300F mutant.

To further investigate the difference in phosphorylation between HisDeaf1 and the Y300F mutant, two-dimensional SDS-PAGE was performed (Figure 2). The removal of a phosphate group would cause a rightward shift toward a higher pH value. Blots did not show a clear and net shift of Y300F mutant compared to Deaf1 in either interval of pH values. Thus, we were unable to detect basal tyrosine phosphorylation of Deaf1 using this approach.

Recombinant HisDeaf1 and the Y300F mutant, HEK293 cells were transfected with HisDEAF1 or the Y300F mutant and extracted for 2D-gel electrophoresis using IEF buffer (see Section 4). The horizontal dimension corresponds to the isoelectric point in pH units. Strips with pH 3 to 10 and 4.7 to 5.9 were used (left and right blots). The vertical dimension is molecular mass determined using the ColorPlus Prestained Protein Ladder, Broad Range (NEB). The position of 80 kDa is indicated by a black bar on the left side of each image.

### 2.2. Effect of GSK-3 Inhibitor Lithium on Deaf1-Mediated Repression of the 5-HT1A Promoter

**Figure 3:**
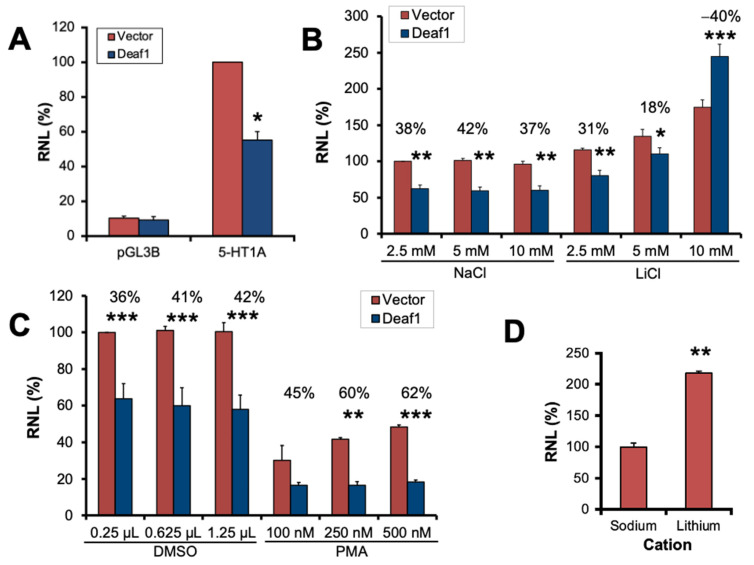
Effect of Lithium or PMA on Deaf1 activity at the 5-HT1A promoter in HEK293 cells. (**A**) 5-HT1A promoter regulation by Deaf1 in HEK293 cells. pGL3B-luciferase reporter without or with the 5-HT1A promoter (5-HT1A) was cotransfected with pcDNA3 without (Vector) or with Deaf1 coding sequence. Units are relative normalized luminescence (RNL) normalized to 5-HT1A-Vector and shown as mean ± SEM; * *p* < 0.05 compared to Vector control. (**B**,**C**) Drug treatments: Cells were transfected with 5-HT1A-luciferase, pcDNA3 without or with HisDeaf1, and pCMV-β-gal. Data are plotted as mean ± SEM of 3 independent experiments each. (**B**) Assays with the indicated concentration (mM) of lithium (LiCl, GSK3β inhibitor) or sodium chloride (NaCl) as osmotic control; RNL was normalized to 2.5 mM NaCl vector control. (**C**) Assays with PMA (PKC activator) or vehicle (DMSO) treatment; RNL was normalized to 2.5 µL DMSO control. The RNL values of all samples were compared by two-way ANOVA followed by Bonferroni-adjusted pairwise comparisons to find *p* values: * *p* < 0.05, ** *p* < 0.01, *** *p* < 0.001. (**D**) Deaf1-independent enhancer activity of lithium. Mouse embryo fibroblast cells from Deaf1 knockout mice were transfected with the 5-HT1A promoter construct and empty Deaf1 vector and treated with 10 mM LiCl or NaCl. ** *p* < 0.01.

We addressed whether GSK-3 inhibition would affect Deaf1 activity. The transcriptional activity of HisDeaf1 at the 5-HT1A promoter was determined using luciferase reporter gene assays (Figure 3A). Compared to the pGL3B empty vector where Deaf1 had no effect, Deaf1 significantly reduced activity of the 5-HT1A promoter by 40% (Figure 3A). The effect on Deaf1 activity of increasing concentrations of the GSK3 inhibitor lithium chloride (LiCl) was tested, with sodium chloride as an osmotic control (Figure 3B). An analysis showed significant differences between control (NaCl) and LiCl treatments (F_(1,24)_ = 201.43, *p* < 0.0005). Moreover, the effect of lithium was concentration-dependent (F_(2,24)_ = 60.28, *p* < 0.0005). Post hoc analysis revealed no significant variation in the Deaf1 effect for matching NaCl concentrations. LiCl treatments displayed a significant increase in 5-HT1A transcriptional activity in the presence of HisDeaf1 between the 2.5 mM to the 5 mM (*p* < 0.05), and between the 5 mM to the 10 mM dose (*p* < 0.0005). Furthermore, while at 2.5 mM and 5 mM of LiCl, HisDeaf1 shows a significant repression relative to pCDNA3 (*p* < 0.005 and *p* < 0.05, respectively); at 10 mM of LiCl, HisDeaf1 showed a significantly increased luciferase activity relative to pCDNA3 (*p* < 0.0005). Overall, these results suggest that the inhibition of GSK3β causes a reversal of Deaf1’s repressor activity at the 5-HT1A promoter.

Since there are also several putative protein kinase C (PKC) sites on Deaf1, we tested whether PKC activation could modulate Deaf1 activity using phorbol 12-myristate 13-acetate (PMA) to activate PKC (Figure 3C). There was a significant repression of the 5-HT1A promoter by HisDeaf1 (F_(1,24)_ = 109.02, *p* < 0.0005) in every case, except for 100 nM PMA (*p* = 0.075). There was a drug effect (F_(1,24)_ = 298.04, *p* < 0.0005) that was a global decrease in normalized RLU values between the vehicle DMSO and the PMA treatments (*p* < 0.0005) and did not depend on PMA concentration (F_(2,24)_ = 0.50, *p* = 0.616). Thus, although PKC affected 5-HT1A promoter activity, it did not affect Deaf1-dependent repressor activity at the promoter.

A slight increase in basal 5-HT1A promoter activity with pCDNA3 control was observed between the 5 mM to 10 mM Li+ dose (*p* < 0.01) (Figure 3B), which may reflect Li action on endogenous Deaf1 or other transcription regulators. We used a line of mouse embryonic fibroblast cells [43] previously generated by our lab from Deaf1 knockout mice [31] to test this possibility (Figure 3D). A dose of 10 mM lithium significantly enhanced 5-HT1A promoter activity in cells transfected with the empty Deaf1 vector condition compared to equimolar sodium chloride (*p* < 0.01), confirming that lithium causes Deaf1-independent enhancement of the 5-HT1A promoter in vitro.

### 2.3. Actions of Selective GSK3β Inhibitors on Deaf1-Mediated Repression of the 5-HT1A Promoter

**Figure 4:**
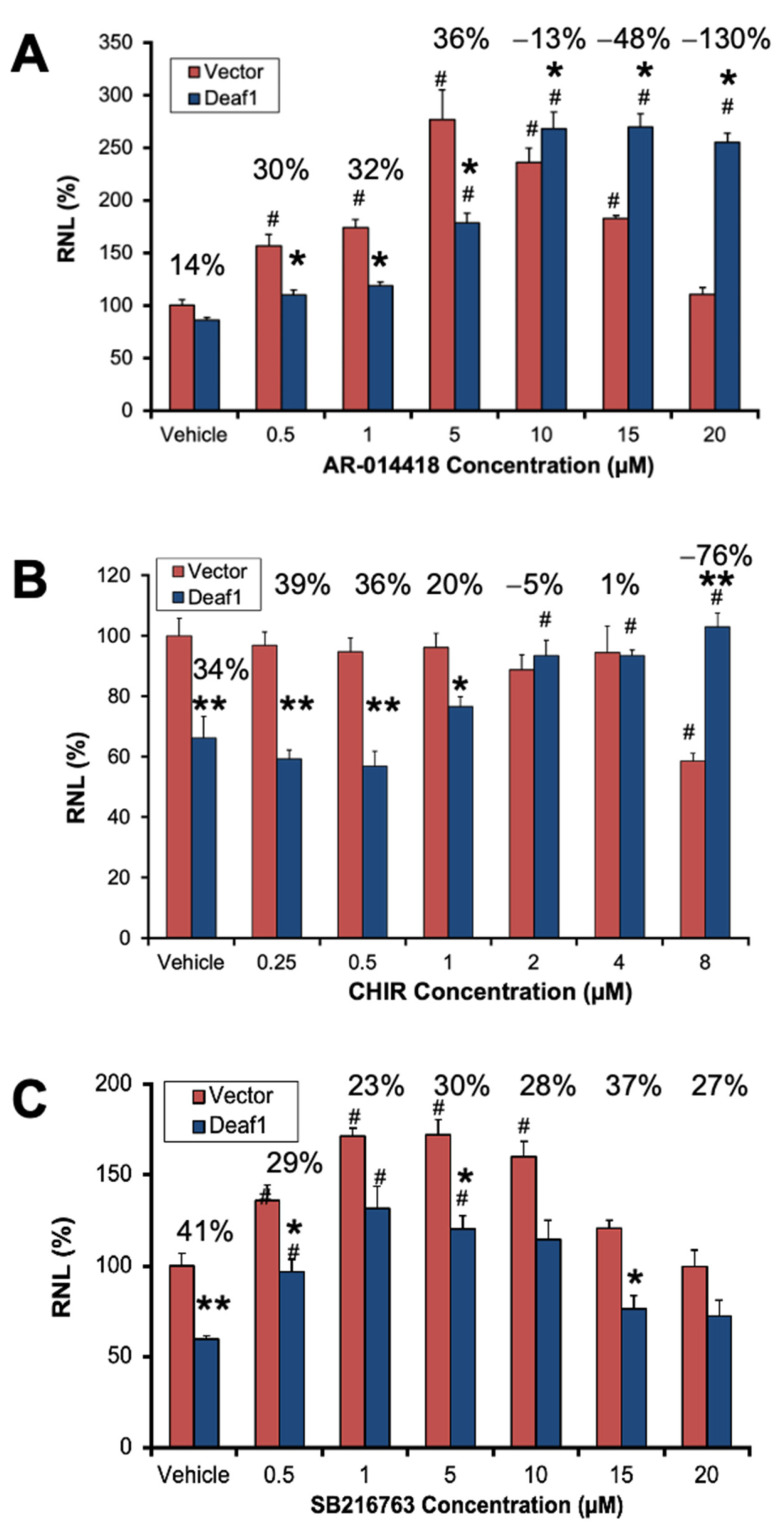
Effect of GSK3β inhibitors on Deaf1 activity at the 5-HT1A promoter. (**A**) AR014418. Effect of AR on the 5-HT1A promoter in the presence and absence of overexpressed Deaf1. Significant concentration × plasmid interaction (F_(1,49)_ = 20.2, *p* < 0.001, R^2^ = 0.47). (**B**) CHIR-99021 (CHIR). Effect of CHIR on the 5-HT1A promoter in the presence and absence of overexpressed Deaf1. Significant concentration × plasmid interaction (F_(1,51)_ = 73.6, *p* < 0.001, R^2^ = 0.63). (**C**) SB216763 (SB). Effect of SB on the 5-HT1A promoter in the presence and absence of overexpressed Deaf1. Concentration × plasmid interaction (F_(1,57)_ = 0.50, *p* = 0.48, R^2^ = 0.40). Data are presented as mean ± SEM, *n* = 3–4. * *p* < 0.05, ** *p* < 0.01 compared to paired vector; ^#^ *p* < 0.05 compared to respective vehicle control. The % repression is shown numerically above each concentration.

Lithium had clear effects on Deaf1 activity, but it is difficult to make a causal link to GSK3β inhibition because lithium has many other targets. To provide further evidence, the concentration-dependent effects of three different GSK3β inhibitors, AR-014418, CHIR-99021, and SB216763 [44], on Deaf1-induced repression of the 5-HT1A promoter were tested (Figure 4). AR-014418 (AR) is a specific inhibitor of GSK3β that has been shown to have antidepressant-like effects in the forced-swim test [45]. In the 5-HT1A promoter assay, AR enhanced activity in both vector and His-Deaf1 conditions (main effect of concentration; F_(1,49)_ = 43.2, *p* < 0.001) and also attenuated repression by Deaf1 (concentration x plasmid interaction; F_(1,49)_ = 20.2, *p* < 0.001) (Figure 3A). Like lithium, AR reversed Deaf1 repression, leading to an enhancer effect compared to pCDNA vector control. Like lithium and AR, CHIR significantly attenuated Deaf1-induced repression (concentration × plasmid interaction; F_(1,51)_ = 73.6, *p* < 0.001). Interestingly, CHIR also reduced basal 5-HT1A promoter activity at the highest concentration, leading to a Deaf1-induced enhancer-like effect as observed with AR. While Deaf1 repressed 5-HT1A promoter activity normally in these experiments (main effect of plasmid; F_(1,57)_ = 22.1, *p* < 0.001), no effect of SB was observed on 5-HT1A promoter repression by Deaf1 (concentration x plasmid interaction; F_(1,57)_ = 0.50, *p* = 0.48). However, exploratory post hoc analysis showed a loss of significant Deaf1-induced repression at higher concentrations of SB, suggesting a partial effect. Taken together, these results suggest that GSK3β inhibitors can reduce Deaf1-induce repression of the 5-HT1A promoter, and even reverse this effect to reveal Deaf1-induced enhancer activity.

**Figure 5:**
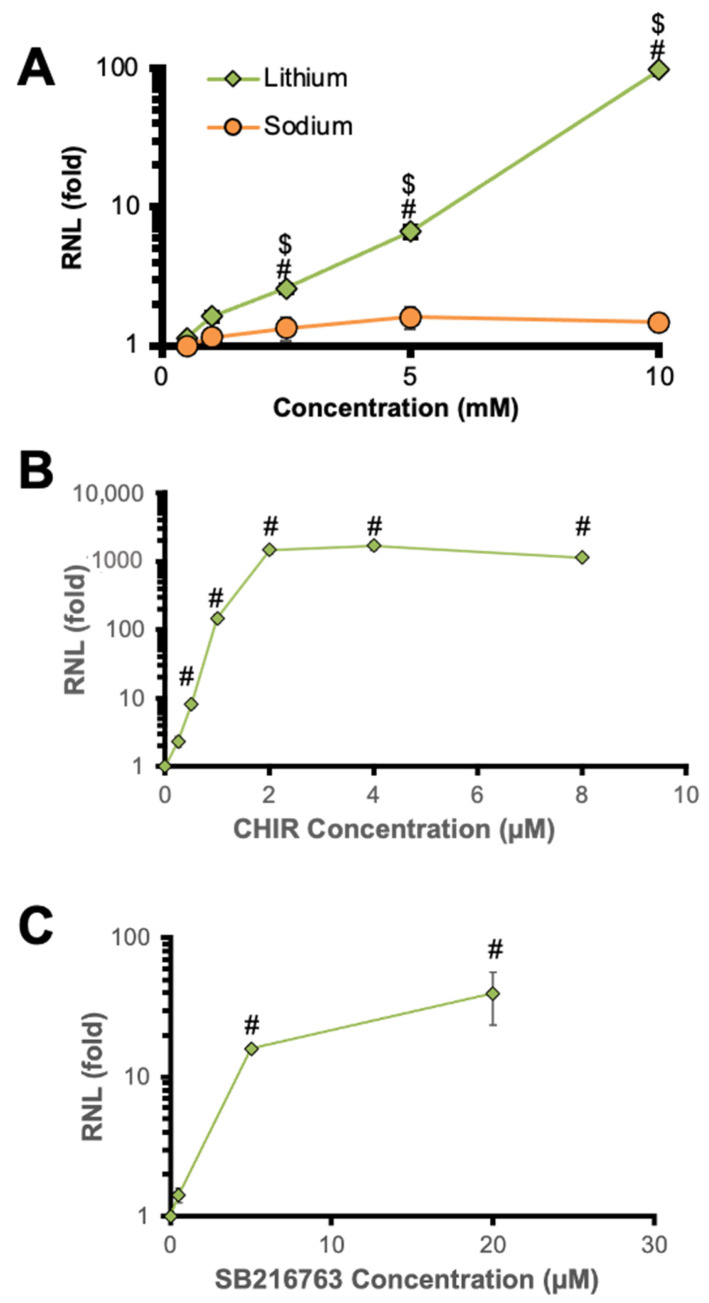
Concentration-dependent inhibition of GSK3β activity on TCF-LEF promoter. Drugs were tested on cells transfected with a TCF/LEF firefly luciferase reporter and relative luciferase activity normalized to Vector control (RNL), with higher RNL representing greater inhibition. Data are plotted as mean ± SEM, *n* = 3. (**A**) Effect of lithium on GSK3β activity. ^#^ *p* < 0.05 compared with the lowest concentration of the same drug; ^$^ *p* < 0.05 for lithium vs. sodium. *n* = 3. (**B**) Effect of CHIR on GSK3β activity. ^#^ *p* < 0.05 vs. vehicle. (**C**) Effect of SB on GSK3β activity. ^#^ *p* < 0.05 compared with vehicle.

To control for the effectiveness of the GSK3β inhibitors, their activity at the GSK3β reporter construct TCF-LEF was tested as a positive control (Figure 5). In this assay, lithium inhibited GSK3β in a concentration-dependent manner (main effect of cation and cation × concentration interaction; F_(1,26)_ = 28.7, *p* < 0.001 and F_(1,26)_ = 10.6, *p* < 0.01, respectively), while sodium had little to no impact (Figure 5A). These results confirm that increasing concentrations of lithium inhibit GSK3β in our conditions, and that this is not due to non-specific effects. Similarly, CHIR potently inhibited GSK3β (main effect of concentration; F_(1,16)_ = 49.6, *p* < 0.001), an effect that was significant at all but the lowest concentration tested in the post hoc (*p* < 0.01 in each case) (Figure 5B). The concentration for maximal effect of CHIR in the TCF-LEF assay (2 µM) matches the maximal inhibition of Deaf1 repression, implicating inhibition of GSK3β as the mechanism. For SB (Figure 5C), a significant concentration-dependent effect was observed (main effect of concentration; F_(1,10)_ = 25.0, *p* < 0.001), indicating its effectiveness in the TCF-LEF reporter assay. Indeed, the post hoc analysis shows that SB has a significant effect on GSK3β activity at concentrations as low at 5 µM (*p* < 0.001). However, the maximal effect of SB was orders of magnitude less than for AR-014418, potentially explaining the lower efficacy to reduce Deaf1 inhibition (Figure 4C). Overall, these data demonstrate that GSK3β inhibitors reduce Deaf1-mediated repressor activity at the 5-HT1A promoter, and at higher concentrations appear to unveil a Deaf1-dependent enhancement of promoter activity.

## 3. Discussion

### 3.1. Deaf1 Phosphorylation

The finding that GSK3β mediates the phosphorylation of Deaf1 in two of three segments of the protein in vitro [34] lead us to hypothesize that GSK3β phosphorylation might regulate the repressor function of Deaf1. Based on the GSK3β inhibitor results, mutating Deaf1 phosphorylation sites should have blocked the Deaf1 repressor activity. It is possible that other sites were not tested or multiple sites needed to be mutated to prevent GSK3β modulation of Deaf-1 activity. Alternately, the effects of GSK3β inhibitors may be due to actions on the GSK3β–Deaf1 interaction [34] rather than on Deaf1 phosphorylation by GSK3β.

In contrast, the mutation of the Y300 site increased Deaf1 repressor activity at the 5-HT1A promoter in two cell lines, suggesting that Y300 phosphorylation inhibits Deaf1 activity. The Y300 site is located adjacent to the NLS of Deaf1 (Figure 1A) and might reduce the translocation of Deaf1 into the nucleus. Although Y300 phosphorylation was identified by mass spectroscopy [42], our 2D-electrophoresis and pull-down assays may be too insensitive to detect partial phosphorylation at a single site. A search of consensus protein kinase sites using the www.phosphonet.ca platform (accessed on 21 August 2023) revealed vascular endothelial growth factor (VEGF) receptors 1-3 as the top candidates for phosphorylating the Y300 site. VEGF receptors are tyrosine kinase membrane receptors with an intracytoplasmic catalytic domain [46] that could target Deaf1. Importantly, VEGF is required for a response to chronic treatment with fluoxetine in stressed rats and is induced in hippocampal neurons via the activation of 5-HT1A receptors [47,48]. Thus, VEGF may both mediate 5-HT1A action and enhance 5-HT1A expression via Deaf1 phosphorylation.

### 3.2. Functional Interaction of GSK3β and Deaf-1

Our findings are consistent with Deaf1 repressor activity at the 5-HT1A promoter in HEK-293 and SKN-SH neuroblastoma cells. We show that lithium and two selective GSK3 inhibitors AK and CHIR concentration-dependently inhibited Deaf1 repressor activity and revealed a Deaf1-dependent enhancer effect at the highest doses. Overall, these results are consistent with the existence of a GSK3β/Deaf-1/5HT1A pathway, except that SB did not significantly reduce Deaf-1 repression. However, under our conditions, the maximal GSK3β inhibition in the TCF-LEF assay by SB was approximately one order of magnitude lower than that of CHIR at 1 µM (the lowest concentration at which an effect on Deaf-1 repression is observed with this compound) (Figure 5). These data suggest that GSK3β inhibition must reach a certain threshold before effects on 5HT1A promoter activity are observed. Thus, the lack of effect of SB may be because it did not reach this threshold of inhibition. It may be that GSK3β Inhibition alone is not enough, and a phosphatase is also necessary to inhibit Deaf-1. Overall, our results support the hypothesis that Deaf1 activity is regulated by GSK3β.

Perhaps the most intriguing finding was that at the highest concentrations, lithium, AR and CHIR revealed a paradoxical enhancer effect of Deaf1 transfection on the 5-HT1A promoter. At these high concentrations, it may be that GSK3β inhibition is not sufficient and that other targets are involved. However, using transfection of a Myc-DEAF1 construct in HEK293T cells, Pilot-Storck et al. [34] found that Deaf1 enhancer activity was increased by 5 mM Lithium chloride as well as by 1 uM azakenpaullone, another GSK3β inhibitor. Since these compounds all inhibit GSK3β with different mechanisms, it can be argued that by inhibiting GSK3β completely, the Deaf1 repressor function is blocked, revealing the Deaf1-dependent activation of the 5-HT1A promoter. Depending on the promoter, Deaf1 acts as a repressor or enhancer. For example, in vitro studies using the same conditions show that Deaf1 knockout in HEK-293T cells reduces the promoter activity of *B4GAT1* and *UBE2M* genes but induces the *RABEP2* gene [49]. Our studies indicate that GSK3β can modify this function of Deaf1, favoring repressor activity that upon its inhibition may release the Deaf1 enhancer function.

How Deaf1 exerts enhancer or repressor functions remains unclear, but Deaf1 functionally interacts with a variety of transcriptional regulators and signaling proteins in addition to GSK3β. These include the transcription factor LMO4 [50], methyl-binding protein MeCP2 [51], E3 ubiquitin ligase Pellino1 [43] and DNA protein kinase subunit Ku70 [52]. Importantly, the Deaf1-MeCP2 interaction at the mouse and human 5-HT1A promoter prevents MeCP2 enhancer activity and activates Deaf1 repressor activity [51]. Thus, alteration in MeCP2-Deaf1 interaction by the GSK3β-mediated phosphorylation of Deaf1 could lead to increased MeCP2-mediated transcription of the 5-HT1A receptor.

In addition to their physical interaction and regulation of Deaf1 by GSK3β, the functional association of Deaf1 and GSK3β is supported by genetic studies in Drosophila. Deaf1 is required for the GSK3β-mediated repression of glycolytic genes in muscle [53]. This finding indicates that not only is Deaf1 regulated by GSK3β, but that Deaf1 is also required for at least some of the transcriptional actions of GSK3β. In mammals, Deaf1 has been implicated in differential regulation of pre- and post-synaptic 5-HT1A receptor expression. Knockout of Deaf1 in mice upregulated 5-HT1A autoreceptors but down-regulated post-synaptic 5-HT1A receptors, especially in the PFC [31]. Similarly, in normal humans heterozygous for the 5-HT1A Deaf1 element, the G-allele that blocks Deaf1 binding was associated with a reduced expression of 5-HT1A in PFC compared to C-allele [29]. Interestingly, heterozygous depressed subjects showed reduced expression of PFC 5-HT1A receptors at the C-allele, suggesting an inactivation of Deaf1 enhancer activity. One potential mechanism could be increased activity of GSK3β to reduce Deaf1 enhancer activity and favor its repressor activity. Interestingly, five-day stress induced GSK3β expression in the amygdala and PFC but not in raphe [54], suggesting that post-synaptic areas may be more stress-sensitive when reducing 5-HT1A expression. Conversely, environmental enrichment leads to the inactivation of GSK3β in the cortex and hippocampus [55], antidepressant actions that could involve an increase in post-synaptic 5-HT1A receptors.

### 3.3. 5-HT1A-GSK3β Signaling in Hippocampus and Antidepressant Response

Complementary to the role of GSK3β in the repression of 5-HT1A gene transcription, 5-HT1A receptors have been shown to inhibit GSK3β signaling to mediate hippocampal neurogenesis and behavioral improvement. Studies in rodents have addressed the effects of 5-HT1A receptors on GSK3β phosphorylation, which inhibits GSK3β. Like lithium, fluoxetine or selective 5-HT1A agonist 8OH-DPAT induces the phosphorylation of GSK3β in mouse and human Down’s syndrome neural precursor cells in vitro [56]. The treatment of normal male mice with fluoxetine or 8OH-DPAT induced GSK3β phosphorylation in the hippocampus via 5-HT1A receptor activation, which was necessary for the anxiolytic effects on behavior [57], as well as for the 5-HT1A-induced hippocampal neurogenesis [58]. Interestingly, in raphe 5-HT neurons, the loss of GSK3β did not affect the signaling of 5-HT1A autoreceptors, but blocked 5-HT1B signaling, suggesting a cell and receptor specificity of 5-HT1A-GSK3β signaling [59]. Thus, the lithium-induced inhibition of GSK3β to increase hippocampal 5-HT1A expression and signaling may further inhibit GSK3β activity and induce antidepressant actions.

### 3.4. Deaf1-Independent Actions of GSK3β Inhibitors

Lithium enhanced the basal activity of the 5-HT1A promoter through a mechanism independent of Deaf1, since similar results were obtained in MEF cells that lack Deaf1 (Figure 3D). Similarly, both AR and SB produced some enhancement that was not clearly tied to Deaf-1 status, but CHIR caused no such enhancement of the basal 5-HT1A promoter activity even when maximal GSK3β inhibition was achieved. This effect could be mediated by a molecular target that is shared by lithium, AR, and SB, but not CHIR. Fortunately, the latter three have already been screened in parallel against a panel of 359 kinases [50], three of which fulfil these criteria: MST2, PCTK1, and STK16. Because lithium inhibits MST2 by only ~6% at 10 mM in vitro [45], the latter two kinases are the most likely candidates to explain the GSK3β/Deaf-1-independent enhancer activity demonstrated in our results.

### 3.5. Therapeutic Relevance: Connection to 5-HT1A Expression in Bipolar Depression

The stimulatory effect of lithium on 5-HT1A promoter activity at clinically relevant concentrations described in this study is consistent with findings in vivo. In male mice subjected to chronic social defeat stress, a two-week treatment with 2.4 mmol/kg/day lithium increased 5-HT1A RNA expression in the raphe by two-fold and had an anxiolytic effect [60]. Similarly, compared to control subjects, the bipolar patients have reductions in cortical 5-HT1A receptor binding, which were reversed by lithium treatment and associated with an improvement in depressive symptoms [61]. The enhanced 5HT1A promoter activity shown in our assay provides a mechanism through which lithium might produce this effect. However, no effect was observed on raphe 5-HT1A receptor levels. In line with these studies, a recent PET imaging study in subjects with bipolar depression found a strong correlation of low pretreatment 5-HT1A binding (particularly in amygdala, hippocampus, and parahippocampal gyrus, but not dorsal raphe) with a better remission rate following 8-week lithium treatment [62], suggesting that a greater capacity for lithium up-regulation in low 5-HT1A patients might improve treatment outcome.

In summary, we find that GSK3β inhibition by a variety of inhibitors including lithium prevents Deaf1-mediated repression of the 5-HT1A receptor, and at high concentrations can switch Deaf1 to increase 5-HT1A promoter activity. These studies suggest that GSK3β may mediate the brain-region-specific regulation of 5-HT1A receptors by Deaf1 and may account for at least some of the beneficial actions of lithium treatment on behavior.

## 4. Materials and Methods

### 4.1. Materials

Lithium chloride and phorbol 12-myristate 13-acetate (PMA) were purchased from Millipore Sigma (Burlington, MA, USA), while CHIR-99021, SB-216763, and AR-014418 were obtained from Cayman Chemicals (Ann Arbor, MI, USA). Lithium was administered as a 1 M solution in water, while specific inhibitors were administered dissolved in DMSO, with a final DMSO concentration of 0.1% or as indicated. Other reagents were obtained from Thermo Fisher Scientific (Mississauga, ON, Canada) or Sigma, except where noted.

### 4.2. Phosphorylation Site Prediction

The full-length human DEAF1 sequence (NCBI, NP_066288.2) was scanned using phosphorylation site prediction algorithms NetPhos 2.0 [63], NetPhosK 1.0 [64], DisPhos 1.3 [65] and GPS 2.1 [66]. For the first three, the programs yielded a score over 1; for GPS 2.1, scores are given along with respective kinase-specific threshold values. To compare these results with the ones found in the other programs, each score was normalized to its respective threshold value, and all resulting values were presented as fractions of the maximum value. The predicted sites in each algorithm were then sorted by score. For the sake of choosing the sites to investigate, a composite score was calculated for every site by summing the respective scores from each algorithm. Sites deemed of interest were selected on three criteria: detection by multiple programs, presence of the site in a functional domain, and value of composite score.

### 4.3. DNA Constructs

The 5-HT1A promoter construct used in this work was previously known as -1128-luc [25]. The construct consists of 1128 bp of the C allele of the human 5-HT1A promoter from the start site of translation, which thus includes the Deaf1 consensus site, inserted into the pGL3-Basic (Promega, Madison, WI, USA) vector upstream of the Luciferase reporter gene. The HisDEAF1 construct is derived from the NUDR construct in [67], with the addition of a histidine tag cloned into the vector at the 5′ end of the *DEAF1* gene. The tag was cloned in between *Kpn1* and *EcoR1* sites to produce a recombinant his-tagged *DEAF1* gene inserted in the pCDNA3 (Invitrogen, San Diego, CA, USA) vector. Mutations of the recombinant *DEAF1* gene were produced by site-directed mutagenesis of the HisDEAF1 construct using the Stratagene QuickChange II XL site-directed mutagenesis kit (Stratagene, Cedar Creek, TX, USA). Table 1 shows each mutant construct with the corresponding primers used. Mutations at these sites serve to abolish potential phosphorylation. Serine and threonine residues were substituted with alanine, while the tyrosine residue is substituted with phenylalanine. The pCMV-β-gal (Clontech, Mountain View, CA, USA) vector was used as a transfection efficiency control during luciferase reporter gene assays. The TCF/LEF luciferase reporter construct (pGL 4.49 from Promega) was used to measure GSK3β activity. It uses six copies of the TCF/LEF binding site to drive the expression of firefly luciferase. GSK3β phosphorylates β-catenin, targeting it for degradation. When GSK3β is inhibited, β-catenin accumulates and binds TCF/LEF sites to drive gene expression. Increased activity of this construct thus signifies the increased inhibition of GSK3β.

### 4.4. Cell Culture and Transfection Procedures

All cells (HEK-293, SKN-SH and Deaf1−/− MEFs) were grown in Dulbecco’s Modified Eagle’s Medium (DMEM) 319-015-CL (containing 4.5 g/L glucose, glutamine, and without sodium pyruvate) supplemented with 10% fetal bovine serum (Wisent, St-Bruno, QC, Canada) without antibiotics except for MEFs, for which penicillin and streptomycin were added. MEFs were generated from Deaf1−/− mouse fetuses [31]. Briefly, fibroblasts from fetuses (age E13.5–E15.5) were harvested and subsequently immortalized using the pWP TS A58 lentiviral vector. The genotype of the lot of MEFs used was verified by PCR.

Transfections in HEK293 cells or SKN-SH cells were conducted using Lipofectamine 2000 reagent (Invitrogen). For each reaction, 2 μL of reagent was used per 1 μg of plasmid. Cells were plated in 6-well plates and grown for 48 h in Dulbecco’s modified Eagle Medium (DMEM) (Wisent, St-Bruno, QC, Canada) with 10% *v*/*v* heat-inactivated fetal bovine serum at 37 °C in 5% CO_2_. For luciferase assays, each transfection contained 1 μg of the pGL3-Basic construct (empty vector or containing 5-HT1A promoter), 1 μg of the pCDNA3 construct (empty vector, with HisDeaf1 or with Deaf1 mutants), and 1 μg of pCMV-β-gal. In experiments using the GSK3β inhibitor lithium chloride (EMD chemicals, Gibbstown, NJ, USA), equal concentration of NaCl was used as control. All drugs were administered at the time of transfection.

For drug concentration dependence studies, HEK293 cells were transfected using the calcium-phosphate method. Cells were sown onto six well plates at a density of 1.5 × 10^5^ cells/well and transfected the next day. A solution of 0.25 M CaCl_2_ containing 2 ng/µL of each plasmid was prepared. To this, an equal volume of 2× HEPES-buffered saline (280 mM NaCl, 50 mM HEPES, and 1.5 mM NaH_2_PO_4_; pH 7.1) was added drop-by-drop. A total of 200 µL of the resulting solution was added to each well of cells.

MEF cells [43] were transfected using FastFect (Feldan, Quebec, QC, Canada) lipid transfection reagent. Transfections were carried out according to the manufacturer protocol, except that the DNA:FastFect ratio was optimized to 1:2.7. As with HEK293 cells, MEFs were sown onto six well plates at a density of 1.5 × 10^5^ cells/well the day before transfection. A total of 1 µg of each plasmid was used per well.

For all cells, the day after transcription the medium was removed, HBBS + EDTA was used to wash away any remaining transfection mix, and fresh medium was added. Cells were harvested and assayed for luciferase activity one day later, as described below.

### 4.5. Luciferase Reporter Gene Assays

Two days after transfection, cells were washed with phosphate-buffered saline (137 mM NaCl, 2.7 mM KCl, 10 mM Na_2_HPO_4_ and 1.8 mM KH_2_PO_4_; pH 7.4) and then gently agitated in 300 µL of 1× Reporter Lysis Buffer (Promega) for approximately 20 min. The partially lysed cells were harvested and put through two freeze-thaw cycles to ensure complete lysis. Lysate was then centrifuged at 13,500× *g* for 2 min to separate out any particulate matter. The resulting supernatant was used in the luciferase and β-galactosidase assays. Luciferase assays were carried out using 100-µL aliquots of each sample loaded onto opaque, white 96-well plates. For the reading of luciferase activity, an equal volume of luciferase buffer (20 mM tricine, 972 µM MgCO_3_ pentahydrate, 2.4 mM MgSO_4_, 90 µM ethylenediaminetetraacetatic acid, 24.3 mM dithiothreitol, 326 µM coenzyme-A and 527 µM adenosine-5′-triphosphate) containing 5.46 mM luciferin potassium salt (Thermo Fisher Scientific, Mississauga, ON, Canada) substrate was injected, mixed for 2 s, and the luminance read for the next 5 s. The β-galactosidase activity was then measured on the SpectraMax M5 (Molecular Devices, Sunnyvale, CA, USA) spectrophotometer at 570 nm. For HEK293 cells, 2 µL aliquots were used, while for MEFs, the volume was 10 µL. Reactions were carried out in Z-buffer (43.2 mM Na_2_HPO_4_, 28.8mM NaH_2_PO_4_, 7.2mM KCl and 0.72 mM MgSO_4_) containing 0.53 mM chlorophenol red-β-D-galactopyranoside (Millipore Sigma, Burlington, MA, USA) substrate in a final volume of 125 µL. Absorbance at λ = 570 nm was read using a SpectraMax M5 (Molecular Devices) immediately after the last reaction was initiated and again at intervals. Reactions were mixed for 5 s before the initial reading, and for 2 s before each subsequent reading, using chlorophenol red galactopyranoside (CRPG) (Millipore Sigma) as a substrate. Normalized RLU were calculated by normalizing every luciferase activity value with the corresponding β-galactosidase absorbance. The % repression was calculated as 100 × (V − D)/V, where V is the average RLU of cells transfected with pcDNA vector, and D is the average RLU of cells transfected with Deaf1 plasmid.

### 4.6. Two-Dimensional SDS Polyacrylamide Gel Electrophoresis

Proteins were extracted using isoelectric focusing (IEF) buffer (42% urea, 15.2% Thiourea (Sigma), 1% DTT (Thermo Fisher Scientific), 4% CHAPS (Sigma), 0.001% bromophenol blue (Bio-Rad, Hercules, CA, USA), in ddH_2_O). A total of 7 cm IPG strips of pH ranges from 3 to 10 and from 4.7 to 5.9 (Bio-Rad) were rehydrated with 25 μg of protein in IEF buffer overnight. The first dimension (isoelectric focusing) was then conducted on Protean IEF Cell (Bio-Rad). Strips were then incubated in reducing solution (36% urea, 2% SDS, 4% iodoacetamide, 20% glycerol, 25% of 1.5 M Tris pH 8.8, in ddH_2_O), followed by incubation in alkylating solution (36% urea, 2% SDS, 1% DTT, 20% glycerol, 25% of 1.5 M Tris pH 8.8, in ddH_2_O). The second dimension was conducted by SDS-PAGE on an 8% gel, followed by transfer to a nitrocellulose membrane and Western blot.

### 4.7. Statistical Analysis

A two-way ANOVA followed by Bonferroni-adjusted pairwise comparisons was used to compare the mean normalized RLU values in the luciferase reporter gene assays. Statistical analysis was performed with Statistical Package for Social Sciences (SPSS) 10.0 (Somers, NY, USA), and calculated using 95% confidence intervals.

## Figures and Tables

**Table 1:** Primers for Deaf1 mutant constructs. Mutated bases are shown in bold.

Mutant	Sense Primer (from 5′ to 3′)	Anti-Sense Primer (from 5′ to 3′)
S43A	GAGCCGGTGCTG**GC**CAGGGACGAGGAC	GTCCTCGTCCCTG**GC**CAGCACCGGCTC
S48A	CAGGGACGAGGAC**G**CGGAGGAGGACGCAGACTC	GAGTCTGCGTCCTCCTCCG**C**GTCCTCGTCCCTG
S54A	GAGGAGGACGCAGAC**G**CGGAGGCGGAGCGGGAGAC	GTCTCCCGCTCCGCCTCCG**C**GTCTGCGTCCTCCTC
T60A	GAGGCGGAGCGGGAG**G**CGCCGCGGGTCAC	GTGACCCGCGGCG**C**CTCCCGCTCCGCCTC
S176A	ACCCCAGGTCCTCAG**G**CTCCTCCAACCCCTCTG	CAGAGGGGTTGGAGGAG**C**CTGAGGACCTGGGGT
T179A	TCAGTCTCCTCCA**G**CCCCTCTGGCTCCC	GGGAGCCAGAGGGG**C**TGGAGGAGACTGA
Y300F	TCAGGCTTTTTGTGCCTT**T**CAAAAGGCGCAAGAAGG	CCTTCTTGCGCCTTTTG**A**AAGGCACAAAAAGCCTGA
T482A	CAAGCCAAGCATGCCAGC**G**CCTACCGAGAAGCTG	CAGCTTCTCGGTAGG**C**GCTGGCATGCTTGGCTTG

## Data Availability

Not applicable.
